# Impact of Home-Based Management of malaria combined with other community-based interventions: what do we learn from Rwanda?

**DOI:** 10.11604/pamj.2013.14.50.2096

**Published:** 2013-02-05

**Authors:** Manasse Nzayirambaho, Jean De Dieu Bizimana, Robert Jean Freund, Pascal Millet, François-Xavier Merrien, Gilles Potel, Pierre Lombrail

**Affiliations:** 1 National University of Rwanda School of Public Health, Kigali, Rwanda; 2 École des Hautes Etudes en Santé Publique, Rennes, France; 3 Centre René-Labusquière, Université Victor-Segalen Bordeaux-2, Bordeaux, France; 4 Faculté des Sciences Sociales et Politiques, Université de Lausanne, Lausanne, Switzerland; 5 Laboratoire EA 3826 (Thérapeutiques Cliniques et Expérimentales des Infections), UFR Médecine et Techniques Médicales, Université de Nantes, Nantes, France; 6 Laboratoire de Santé Publique et d'Epidémiologie, UFR Médecine et Techniques Médicales, Université de Nantes, Nantes, France

**Keywords:** Children, fever, malaria, Rwanda

## Abstract

**Introduction:**

This study aimed to evaluate the impact of home-based management of malaria (HBM) strategy on time to treatment and reported presumed malaria morbidity in children aged less than 5 years in Rwanda.

**Methods:**

The study was carried out in two malaria-endemic rural districts, one where HBM was applied and the other serving as control. In each district, a sample of mothers was surveyed by questionnaire before (2004) and after (2007) implementation of HBM.

**Results:**

After implementation, we observed: i) an increase (P < 0.001) in the number of febrile children treated within 24 hours of symptom onset in the experimental district (53.7% in 2007 vs 5% in 2004) compared with the control district (28% vs 7.7%); ii) a decrease in the reported number of febrile children in the experimental district (28.7% vs 44.9%, P < 0.01) compared with the control district (45.7% vs 56.5%, P < 0.05).

**Conclusion:**

HBM contributed to decrease time to treatment and reported presumed malaria morbidity.

## Introduction

In sub-Saharan Africa, children less than 5 years of age continue to pay the heaviest tribute to malaria [1, 2]. In response to this situation, many countries have adopted the strategy of home-based management of malaria (HBM) [3] supported by the World Health Organization and have complemented it with other community-based interventions. This is the case in Rwanda, where the introduction of HBM was done in parallel with the community-based health insurance schemes locally known as? mutuelle de santé? [4], the distribution of insecticide-treated bed nets, and the introduction in the health facilities of artemisinin-based combination therapy (ACT) [5, 6]. Studies based on health facility statistics after implementation of these latter two interventions notably showed fewer hospital admissions for severe malaria [5], and reduced malaria morbidity and mortality [6] in children less than 5 years old in the health facilities. These decreases occurred while the development of community-based health insurance schemes was improving access to care in the facilities [4]. The part played by HBM in this decrease has still to be evaluated, taking into account the fact that at the time, treatment was given without rapid diagnostic test (RDT) or laboratory confirmation of malaria.

In Rwanda, the HBM strategy was adopted in May 2004. It aims to facilitate access to care and to reduce the time to effective treatment. In December 2004, the district of Nyanza was one of the first pilot districts where HBM was tested. In this country, HBM is based on voluntary community health workers known as drug distributors (DDs) who are elected by the population and trained for this activity. Currently, DDs use RDT to detect malaria prior to any treatment. However, by the time this study was conducted, DDs were trained to recognize malaria using a simple algorithm and to administer ACT according to an age-appropriate treatment protocol. Any child aged less than 5 years with febrile illness was considered to have malaria. The DDs examining the febrile children brought by their mothers had to decide to address them to the health center or to treat them at home if there was no contra-indication. In the latter case, the DDs sold to the mothers, at a subsidized price, pre-packaged artemether-lumefantrine (AL) tablets, and gave the first dose. A child was considered febrile if the DD personally noted, by touch, the presence of fever, or if the mother reported that the child has had fever.

In addition to distribution of drugs, DDs were also in charge of sensitizing and providing information to the community and in particular to mothers on early consultation for fever and the correct administration of anti-malaria drugs to children aged less than 5 years.

In Africa, studies on the health impact of HBM, including its effects on morbidity, are still sparse, particularly as many countries are changing their malaria treatment policy in favor of ACT [7]. In Rwanda, there has been no study of the health impact of HBM at a community level. Such evaluation is increasingly important as HBM continues to be extended in other African countries.

The aim of this study was to evaluate, at a community level, the impact of HBM on time to treatment and on malaria morbidity (febrile episodes without RDT or laboratory confirmation), and on reported all-cause morbidity in children aged less than 5 years in the pilot district of Nyanza two and a half years after its introduction, independently of the possible impact of other community-based interventions.

## Methods

### Study design

A comparative study was carried out in 2 districts, the HBM pilot district and a control district. The variables studied were measured in both districts 5 months before and 2 and a half years after implementation of HBM. Between these two measurements, HBM was implemented by the national malaria control program. During and after implementation of HBM, all other activities relating to malaria control continued as usual in both districts.

### Setting of the study

The two districts in which the study was carried out are rural and malaria was endemic. The district of Nyanza was chosen by the Ministry of Health as a pilot district for HBM because of its malaria endemicity and the presence of a financial partner. The district of Bugesera was randomly chosen among the malaria-endemic districts where HBM had not yet been introduced. The district of Nyanza is located in the South Province of Rwanda, whereas the district of Bugesera is located in the East Province. In Rwanda, the major malaria vectors are *Anopheles gambiae* and *Anopheles funestus*. Plasmodium falciparum is the predominant species, responsible for over 90% of cases [8, 9]. Like all the country, Nyanza and Bugesera have two principal rainy seasons: between February and April, and between September and November. According to the National Institute of Statistics of Rwanda, in 2007 the district of Nyanza had a population of 239 707 inhabitants and the district of Bugesera 274 113 inhabitants. Children aged less than 5 years represented about 15% of the total population in the two districts. Both districts have a subsistence economy based essentially on agropastoral activities.

### Sampling

The pre-implementation study comprised 362 mothers (178 in the district of Nyanza and 184 in the district of Bugesera) with a participation rate of 98%. Its aim was to collect basic data to be used to evaluate the impact of HBM after implementation. For the post-implementation study, sample size was calculated to include at least 40% of febrile children aged less than 5 years treated within 24 hours of symptom onset in the experimental district (or two times more than in the control district). With a power of 90% and a 95% confidence interval, taking into account a cluster effect of 2 and a non-response rate of 2%, the minimum number of mothers required in the two districts was 376, or 188 in each district. All mothers took part in the study, a participation rate of 100%. During the two studies, before and after implementation, the mothers investigated were recruited in cells (the smallest administrative unit at the time), which were selected by the cluster sampling method. In each cell selected, the number of mothers to be investigated was estimated in proportion to the size of the cell. The investigators visited the households following a circular centrifugal route (starting from the center towards the periphery of the cell) until the required number of persons had been included. In each household, the carers of the children aged less than 5 years were questioned.

### Variables and data collection

The questionnaires were designed to determine, before and after HBM implementation: i) time to treatment for febrile children aged less than 5 years reported by the mothers during the 2 weeks before our survey; ii) the occurrence of fever (presumptive malaria); iii) the development of illness (all disease causes). The questions put were identical before and after implementation. After implementation, specific questions on HBM were added and were put only in the experimental district. The DDs and health facilities (dispensaries, health centers, hospitals) were considered as formal sources of care.

The first study was carried out in July 2004 and the second in July 2007. The questionnaires were administered in the households to the carer of the children aged less than 5 years (the biological mother, or in her absence, another person aged at least 18 years). In this study, « mother » refers to the carer of the sick child in the household. The aims of the study were first explained to the mothers, who were then included in the study after giving their voluntary informed consent. Mothers who were not resident in the district were excluded from the study. The mothers were interviewed face to face and the questionnaires were directly filled in by the investigators.

Experienced investigators were carefully selected and trained in administration of the questionnaire. The questionnaires were translated into kinyarwanda (the native language of Rwanda) and pre-tested before being applied. The completed questionnaires were checked the same day to ensure they were correctly and fully completed. Double data entry was performed using Epi Info 3.3.2 (CDC, Atlanta, GA, USA). Data were converted into SPSS 13.0 (SPSS Inc., Chicago, IL, USA) for statistical analysis. In each district, the variables studied were compared before and after implementation of HBM and the data of the experimental district were compared with those of the control district. Comparisons were made using the chi-squared test and Fisher's exact test for small series, and the conventional level of 5% was considered significant.

## Results

### Characteristics of the sample

The majority of mothers were aged less than 40 years, were married and had an agropastoral activity (94%). One in 5 was illiterate (Table 1). In both districts, at least 90% of mothers recognized fever as a sign of a malaria episode.

**Table 1:** Sociodemographic characteristics of mothers, 2004

Variable	Experimental district	Control district	P
	n = 178 (%)	n = 184 (%)	
**Maternal age, year**			
< 21	7 (3.9)	8 (4.3)	0.546
21 – 30	71 (39.9)	78 (42.4)	
31 – 40	76 (42.7)	82 (44.5)	
> 40	24 (13.5)	16 (8.8)	
**Civil status**			
Married	143 (80.3)	160 (87.0)	0.284
Single	11(6.2)	8 (4.3)	
Widow	17 (9.5)	9 (4.9)	
Separated	7 (4.0)	7 (3.8)	
**Educational level**			
Illiterate	39 (21.9)	36 (19.6)	0.250
Uncompleted primary education	66 (37.1)	84 (45.6)	
Completed primary education and more	73 (41.0)	64 (34.8)	

### Time to treatment

After implementation of HBM, the percentage of febrile children aged less than 5 years who were treated within 24 hours improved significantly in both districts (PTable 2). In this experimental district, the majority of febrile children were treated in a formal health care source (35/54), by a DD (26/35), and within 24 hours (23/26). Lastly, among the febrile children who had not received treatment within 24 hours (other treatment modalities), 19/25 had not been treated in a formal health care source. Also, if the anti-malaria treatment given was ineffective, the DDs referred the sick children to the nearest health facility.

**Table 2:** Time to treatment of febrile children aged less than 5 years, n (%)

Time to treatment in previous 2 weeks	Experimental district			Control district		
	Before HBM (n = 80)	After HBM (n = 54)	*P*	Before HBM (n = 104)	After HBM (n = 86)	*P*
Treated within 24 h in a formal healthcare source (health facility or DD)	4 (5.0)	29 (53.7)	< 0.001	8 (7.7)	24 (28.0)	< 0.001
Other modalities	76 (95.0)	25 (46.3)		96 (92.3)	62 (72.0)	

HBM: home-based management of malaria; Health facility: dispensary, health center, hospital; DD: drug distributor

### Malaria morbidity and overall reported morbidity in the community

After implementation of HBM, the reported proportion of febrile children among the children aged less than 5 years (presumed malaria morbidity) decreased in both districts, experimental (28.7% in 2007 vs 44.9% in 2004, (Table 3).

**Table 3:** Reported malaria morbidity and general morbidity in children aged less than 5 years, n (%)

	Experimental district			Control district		
	Before HBM (n= 178)	After HBM (n= 188)	*P*	Before HBM (n= 184)	After HBM (n= 188)	*P*
**Malaria morbidity (febrile children in previous 2 weeks)**						
Febrile children	80 (44.9)	54 (28.7)	< 0.01	104 (56.5)	86 (45.7)	< 0.05
**General morbidity (sick children in previous 2 weeks)**						
Sick children (all disease causes)	106 (59.5)	101 (53.7)	0.260	123 (66.9)	121 (64.4)	0.613

HBM: home-based management of malaria

## Discussion

### Time to treatment

Rapid treatment of malaria is critical as it can progress to severe disease in a few hours, especially in children [10]. In the study carried out before HBM implementation, we observed that in both districts (experimental and control), less than 1 in 10 children aged less than 5 years was taken to a formal health care source within 24 hours. Similar results have been reported in other malaria-endemic countries [11].

Two and a half years after HBM implementation, we observed that time to treatment had decreased in both districts. This is probably related in particular to improved financial accessibility of care thanks to the community-based health insurance schemes. At a national level, the membership rate of this health insurance system in fact rose from 27% in 2004 to 75% in 2007[12]. A study assessing the impact of community-based health insurance schemes on access to care in Rwanda [4] showed that members made greater use of health services than non-members (4 to 8 times more for curative consultation).

However, our results have shown that after implementation of HBM, time to treatment was shorter in the experimental district. In this district, half the children aged less than 5 years (compared with less than 1 in 3 children in the control district) were taken to a formal health care source within 24 hours of fever onset, and in 8 of 10 cases they were taken to a DD. This suggests that HBM had a specific impact on the number of children treated within 24 hours. The results of our study confirmed those of the health facility reports [13] showing that, in HBM areas, 80% of febrile children aged less than 5 years at the time seen by DDs and in health centers were treated within 24 hours of symptom onset. An evaluation of HBM in Uganda [14] had also shown that in 2003, 56% of child carers sought treatment within 24 hours of symptom onset after the introduction of HBM.

### Reported malaria morbidity in the community

Our results suggested that implementation of HBM was followed by a significant decrease in the number of febrile children aged less than 5 years in the two districts. This decrease may be due to the combined effect of several interventions. In addition to introduction of the community-based health insurance scheme, insecticide-treated bed nets had been distributed on a nation-wide scale to children aged less than 5 years and pregnant women during the observation period. During the same period, in all health facilities in the country the Ministry of Health introduced AL as first-line anti-malaria treatment instead of amodiaquine and sulfadoxine-pyrimethamine.

Studies have shown that cases of pediatric malaria seen in the health facilities decreased following the distribution of insecticide-treated bed nets [15] or introduction of ACT in health facilities [16]. Our results suggested, however, that malaria morbidity (in fact, febrile episodes) decreased more in the experimental district. This could be explained by the preventive effect of early anti-malariatreatment leading to a reduced level of transmission.

Few studies have found that programs for treatment of presumptive malaria in children aged less than 5 years by community health workers can reduce malaria morbidity [17, 18]. A systematic review of publications on the health impact of HBM in Africa [7] concluded that the efficacy of the HBM strategy, in particular in reducing malaria morbidity, remains to be proven. This is especially important as the fear that parasite resistance may be induced by widespread use of antimalarials has not been lifted [19].

Not all febrile children treated presumptively by DDs necessarily had malaria, for fever is a symptom of many other diseases, including respiratory tract infections. By the time this study was conducted, DDs did not have the possibility of confirming the presence or absence of the parasite in children reported to be « febrile » by their mothers. However, we assume that the situation in Rwanda probably did not differ from that of the other malaria-endemic African countries, where the majority of cases of fever were malarial [20].

## Conclusion

Our findings suggested that implementation of HBM led to a significantly higher proportion of febrile children receiving treatment within 24 hours of symptom onset and to a decrease in reported malaria morbidity. These effects were accompanied by a general trend towards improvement, probably attributable in particular to the development of community-based health insurance schemes, the distribution of insecticide-treated bed nets and the introduction of ACT in the health facilities.
